# Evaluating the validity of lightweight talar replacement designs: rational models and topologically optimized models

**DOI:** 10.1186/s40824-022-00256-8

**Published:** 2022-03-14

**Authors:** Yeokyung Kang, Seongjin Kim, Jungsung Kim, Jin Woo Lee, Jong-Chul Park

**Affiliations:** 1 Central Research & Development Center, Corentec Company, Limited, Seoul, Republic of Korea; 2 Cellbiocontrol Laboratory, Department of Medical Engineering, Yonsei University College of Medicine, Seoul, Republic of Korea; 3 Department of Orthopaedic Surgery, Yonsei University College of Medicine, Seoul, Republic of Korea

**Keywords:** Talar replacement, Topology optimization, Finite element analysis, Rational scaffold, Optimized scaffold

## Abstract

**Background:**

Total talar replacement is normally stable and satisfactory. We studied a rational scaffold talus model for each size range created through topology optimization (TO) and comparatively evaluated a topologically optimized scaffold bone talus model using a finite element analysis (FEA). We hypothesized that the rational scaffold would be more effective for application to the actual model than the topologically optimized scaffold.

**Methods:**

Size specification for the rational model was performed via TO and inner scaffold simplification. The load condition for worst-case selection reflected the peak point according to the ground reaction force tendency, and the load directions “plantar 10°” (P10), “dorsi 5°” (D5), and “dorsi 10°” (D10) were applied to select worst-case scenarios among the P10, D5, and D10 positions (total nine ranges) of respective size specifications. FEA was performed on each representative specification-standard model, reflecting a load of 5340 N. Among the small bone models selected as the worst-case, an arbitrary size was selected, and the validity of the standard model was evaluated. The standard model was applied to the rational structure during validity evaluation, and the TO model reflecting the internal structure derived by the TO of the arbitrary model was implemented.

**Result:**

In worst-case selection, the highest peak von Mises stress (PVMS) was calculated from the minimum D5 model (532.11 MPa). Thereafter, FEA revealed peak von Mises stress levels of 218.01 MPa and 565.35 MPa in the rational and topologically optimized models, respectively, confirming that the rational model yielded lower peak von Mises stress. The weight of the minimum model was reduced from 1106 g to 965.4 g after weight reduction through rational scaffold application.

**Conclusion:**

The rational inner-scaffold-design method is safer than topologically optimized scaffold design, and three types of rational scaffold, according to each size range, confirmed that all sizes of the talus within the anatomical dimension could be covered, which was a valid result in the total talar replacement design. Accordingly, we conclude that an implant design meeting the clinical design requirements, including patient customization, weight reduction, and mechanical stability, should be possible by applying a rational inner scaffold without performing TO design. The scaffold model weight was lower than that of the solid model, and the safety was also verified through FEA.

## Background

The talus forms the ankle joint and is located superiorly in the foot. It is located inferior to the tibia and fibula, supporting both bones, and is responsible for transferring weight to the feet. Moreover, the talus is covered in cartilage along with the fibula and other ankle bones to control feet movement. Since 60% of the talus is covered by cartilage and surrounding bones, blood supply is poor, and avascular necrosis (AVN) occurs frequently [1, 2]; 75% of cases of AVN of the talus are caused by trauma, and 25% have nontraumatic etiologies, including polycythemia [3, 4]. Currently, for AVN of the talus, conservative treatment is preferred over surgical treatment, and surgical treatment, such as talectomy and talus fusion, is performed when pain is severe or walking is impossible. Talectomy is no longer recommended as it leads to poor functional results, shortening of the lower extremities, and marked postoperative destruction of the calcaneus. When the damage to the existing talus bone is severe, talus fusion is performed, removing the existing talus and implanting a bone that is fixed in the ankle joint. Because the postoperative ankle joint is immobile, the patient’s gait is not natural and the load on the adjacent joint is heavy; patients prefer not to undergo such treatment, as the surgical outcome is not much better than with talectomy [5].

Ankle joint surgery that can be replaced includes total talus arthroplasty and total ankle arthroplasty. However, total ankle arthroplasty has a disadvantage in that the talus, as well as the tibia, where it is in contact with the talus, must be excised and replaced with an artificial joint, even when the tibia is intact. In contrast, total talus arthroplasty replaces only the talus, maintaining the limb length [1]. Moreover, total ankle arthroplasty is contraindicated in AVN patients [6]; in such patients, only the talus should be replaced with an implant with proven safety. Moreover, as the need for implants rises because of an increase in demand related to talar idiopathic AVN and trauma [1], total talar arthroplasty is expected to show superior results compared to total ankle arthroplasty for quick pain relief. To perform such total talus arthroplasty, the customized talus implants are generally manufactured using the powder bed fusion three-dimensional (3D) printing method. The first advantage of a talus implant manufactured using 3D printing is that an anatomically fitting ankle can be reconstructed using the patient’s normal contralateral talus as a template for the design, reducing postoperative discomfort and increasing ankle function [7]. The second advantage is that, with topology optimization, a lightweight talus implant can be printed. In previous machine-manufactured implants with filled-in inner spaces, the weight of the materials constituting the implant could cause discomfort, and there was a limit to the customization of the shape of the implant. With 3D printing, a form that cannot be manufactured by a machine can be achieved, and an optimal, lightweight implant can be manufactured. Moreover, 3D printing can be used to manufacture an implant that eliminates unnecessary weight from the implant interior; furthermore, improved structural strength can be achieved via topology optimization [8].

There have been several reported cases of total talus replacement since the development of third-generation, patient-customized talus implants based on the technological advancements of 3D printing. A case series was reported by researchers from Duke University (Durham, NC, US), including 27 patients with external trauma to the talus. The surgery was performed using an anterior approach with 3D-printed titanium or cobalt-chromium talus implants. In their report, patients were satisfied with the outcomes after a mean follow-up of 22.2 months postoperatively, reported no complications, and showed an increase in American Orthopaedic Foot and Ankle Society score from 47.7, preoperatively, to 78.2, postoperatively [9]. Therefore, total talus replacement is a surgical technique associated with high patient satisfaction and stability. Moreover, they demonstrated that using customized implants, ankle-cartilage motility can be preserved, and preoperative lower extremity length can be maintained, improving the ankle-cartilage function score of the patient [9].

This process shortens the implant manufacturing time compared to the time needed to manufacture existing, conventional talus replacement implants, creates a product that is as lightweight as possible, and creates a safer scaffold. Thus, our study aimed to compare the rational model comprising a three-type scaffold (minimum, medium, and maximum), integrated into the internal structure for each size range, with a topologically optimized scaffold, integrated according to each internal structure size before the development of total talus replacement for commercialization. This study indicates that the rational-model scaffold for which the default shape is specified according to size (minimum, medium, and maximum) is a safer and more effective application of the design, and leads to less time needed than that required for the actual model, than the topologically optimized model scaffold, which requires optimization for every model.

## Methods

**Fig. 1:**
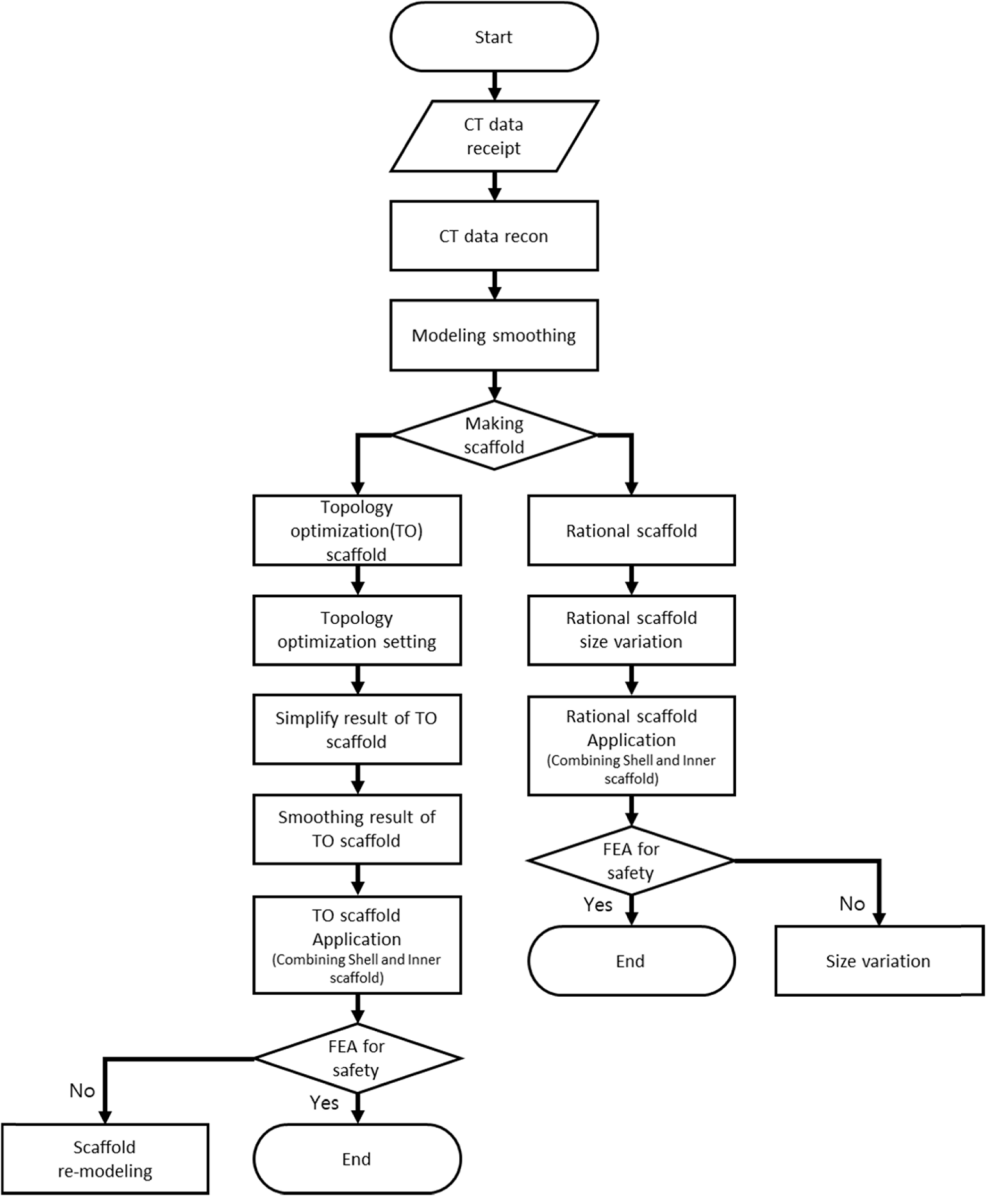
Flow chart of the diagram of the whole process with different models (TO scaffold and rational scaffold)

Figure 1 shows the flowchart for the whole process, from modeling of the total talus implant to applying the inner scaffold. In this process, we compared the rational scaffold design procedure according to the size classification of the talus implant with the model that forms a scaffold by applying topology optimization to each model, and introduced a safety comparison method.

### Selecting the specification range for total talus implant

**Fig. 2:**
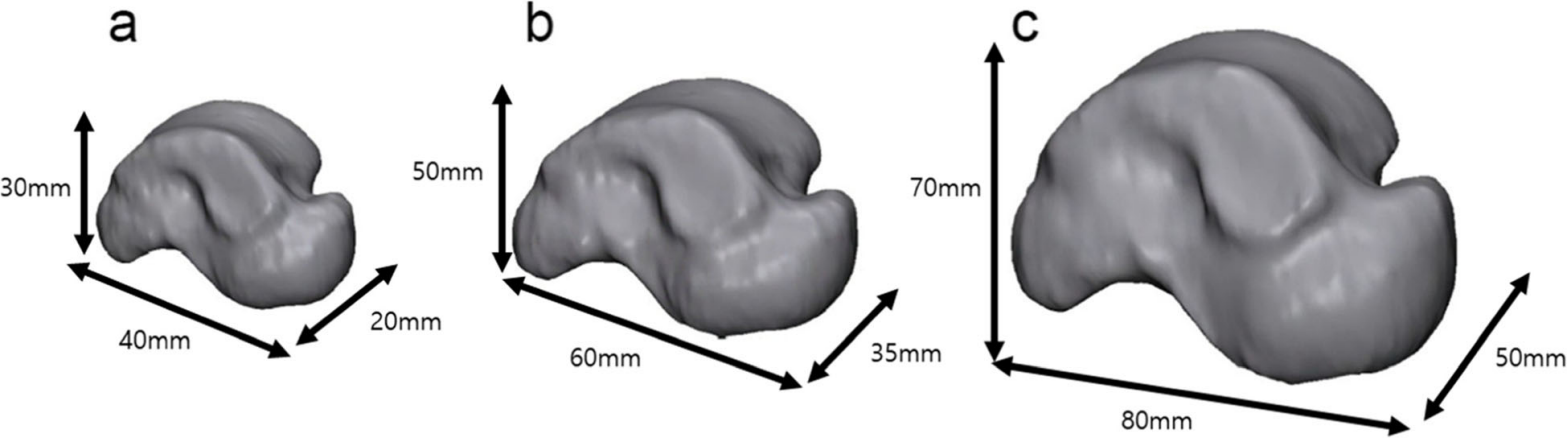
Three-dimensional reconstruction models of the representative specification. (**a**) Minimum model, (**b**) medium model, and (**c**) maximum model

The rational model for each specification of talus implant was established by 3D bone model restructuring, topology optimization, and design simplification. The specification range of the implant was divided into small, medium, and large sizes (Table 1), considering the actual anatomical sizes. The representative model for each specification range was selected considering the length, width, and height of each implant (Table 2), with the smallest sizes in the small range, medium sizes in the medium range, and largest sizes in the large range, as a worst-case model [10–15]. The selected model was restructured based on data obtained with computed tomography of a cadaver, using Mimics software (Materialise NV, Leuven, Belgium) (Fig. 2).

**Table 1:**
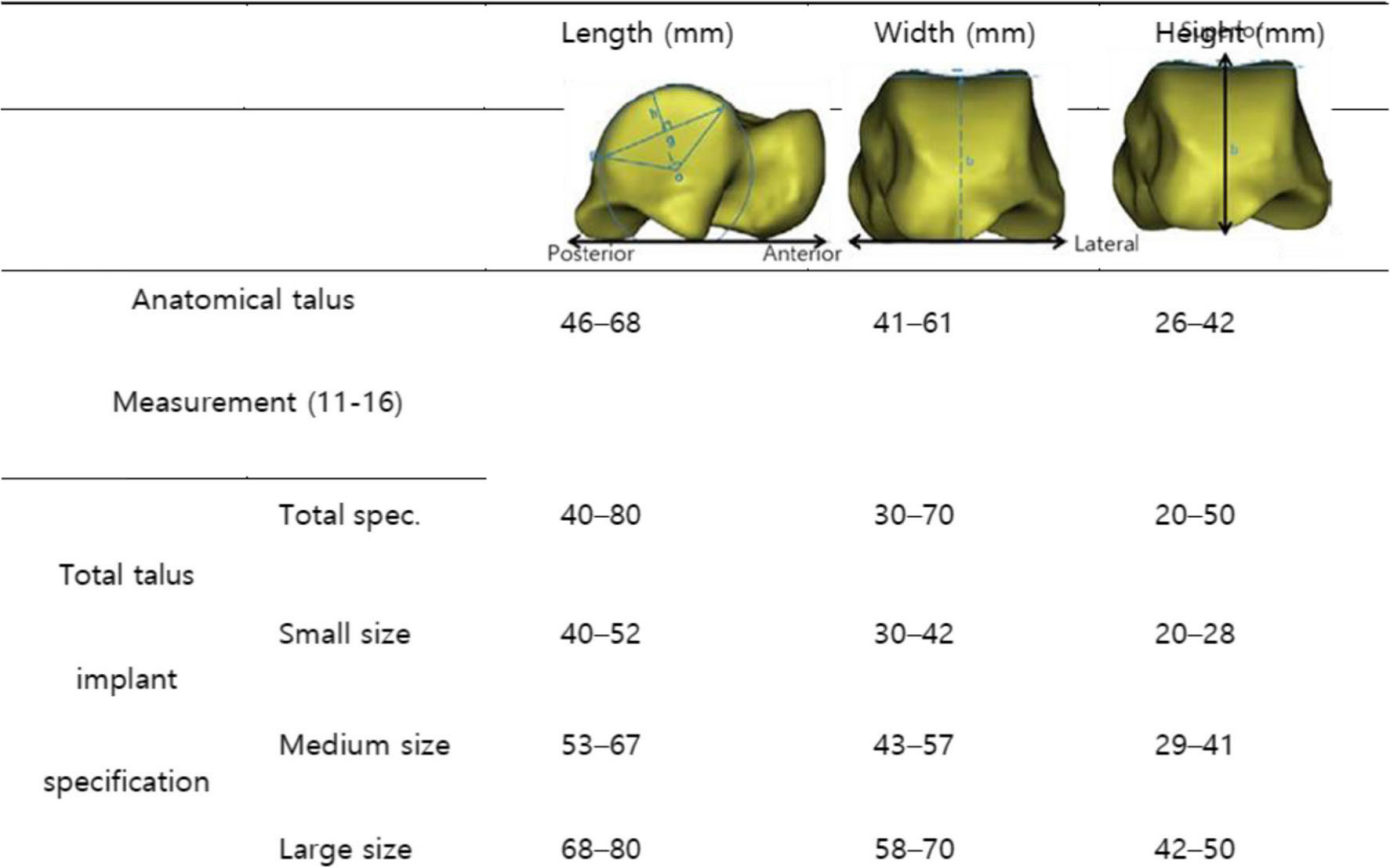
Anatomical dimensions of the talus bone and specification range of the total talus implant

**Table 2:** The representative models of each specification range in total talus implant

Implant specification	Length (mm)	Width (mm)	Height (mm)
Minimum model (small size)	40	30	20
Medium model (medium size)	60	50	35
Maximum model (large size)	80	70	50

#### Topology optimization-based inner structure design

**Fig. 3:**
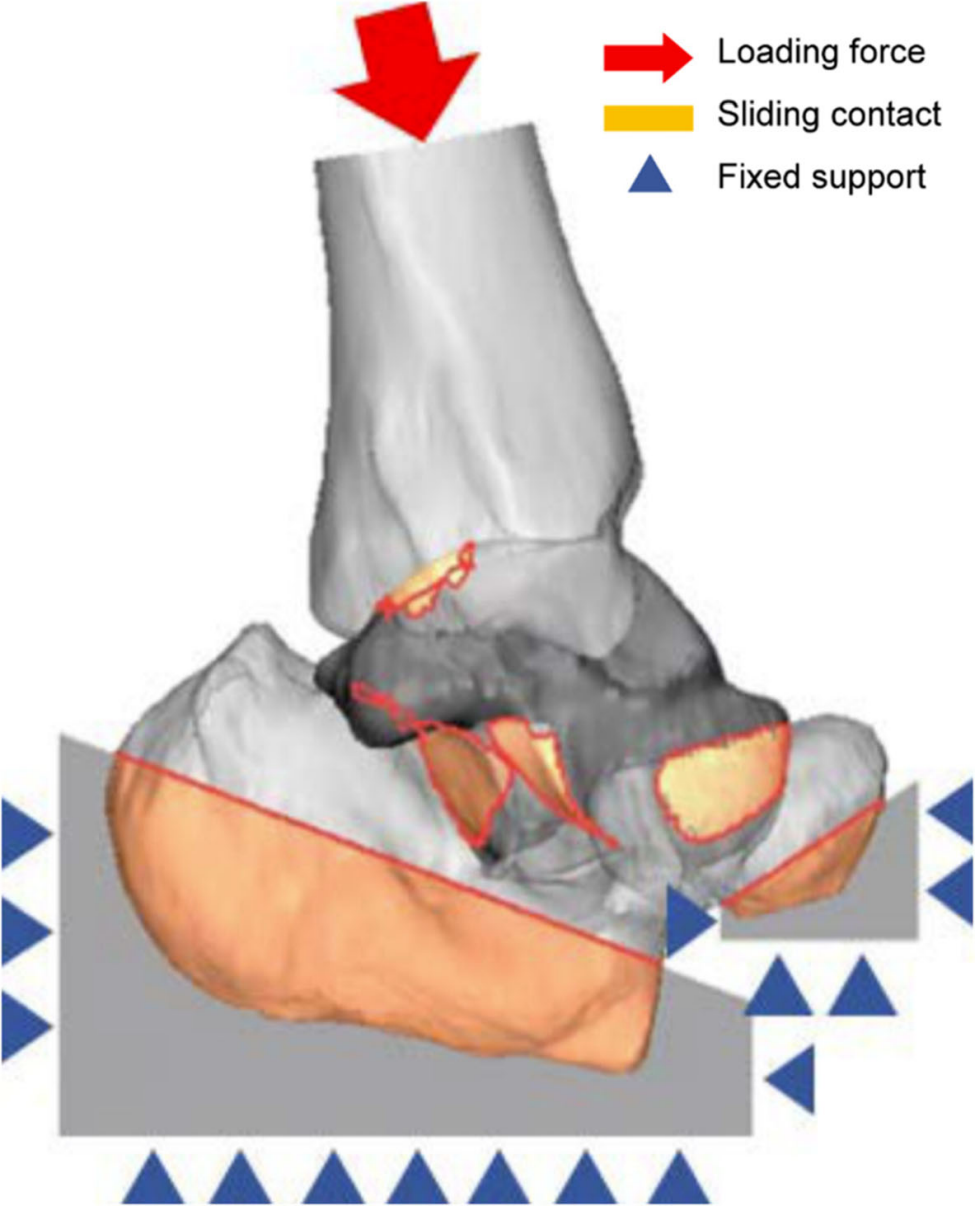
Boundary and loading conditions of topology optimization for inner scaffold

**Fig. 4:**
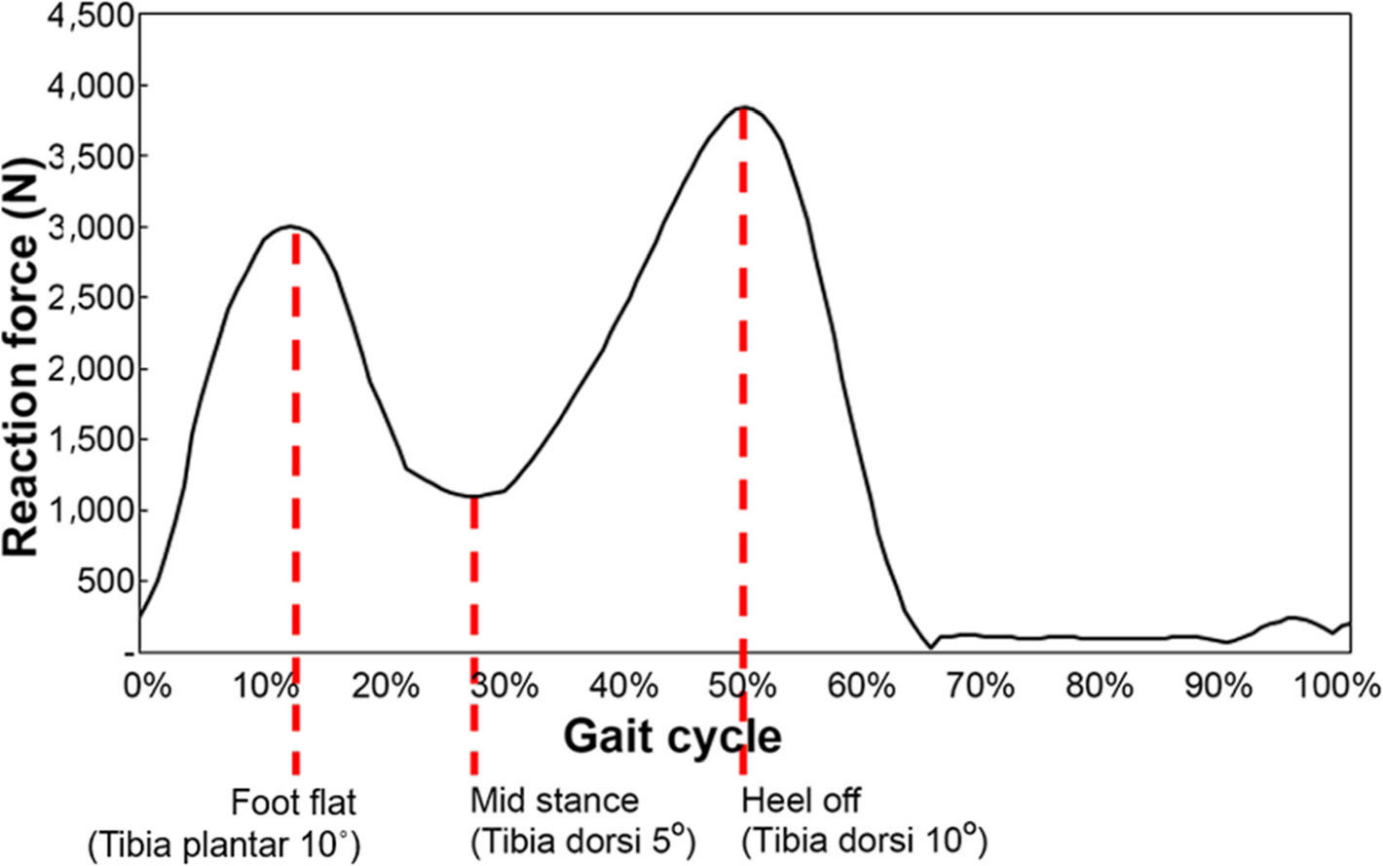
Reaction force point in gait cycle graph

**Fig. 5:**
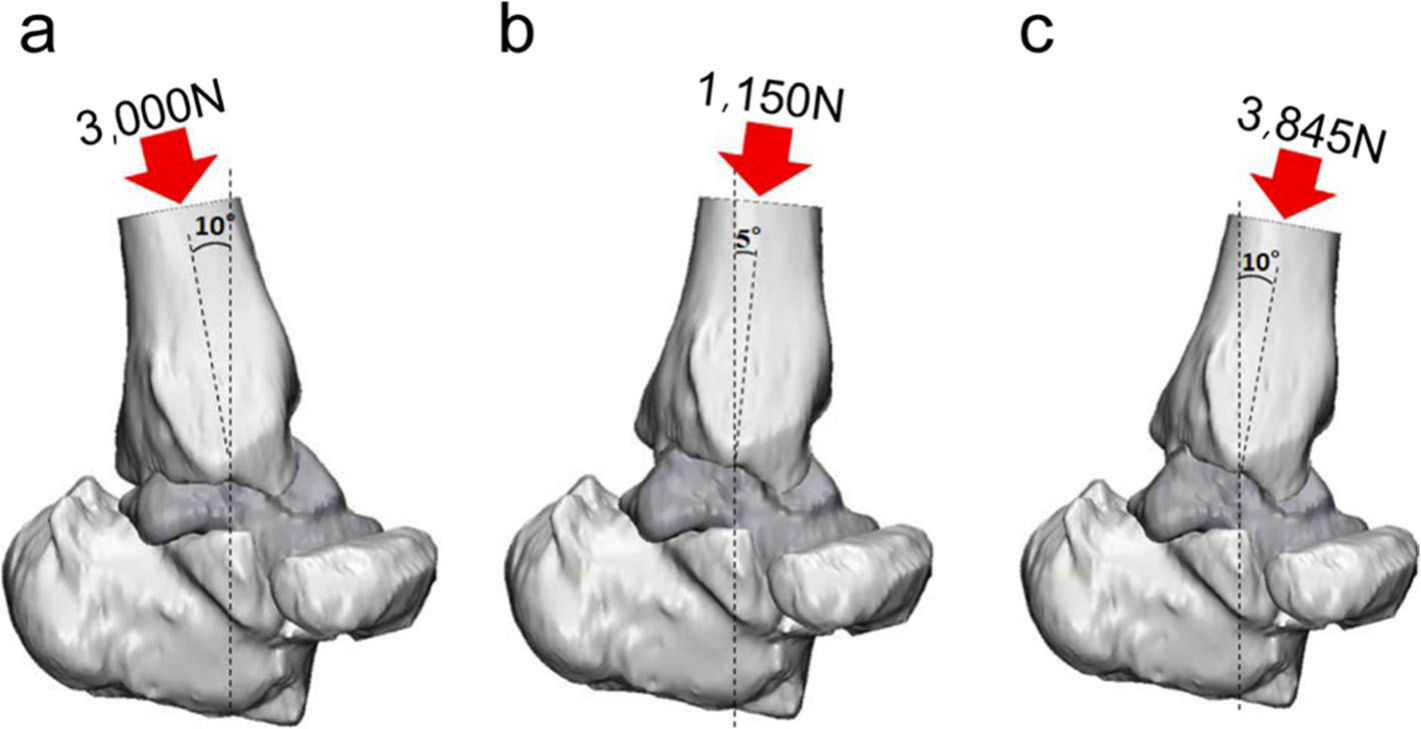
Loading conditions based on each peak point in gait cycle (**a**) foot-flat (plantar 10°), (**b**) mid-stance (dorsi 5°), and (**c**) heel-off (dorsi 10°)

For the topology optimization of the load-bearing inner structure, Inspire solidThinking software (Altair Engineering, Inc., US) was used. The distal tibia, calcaneus, and navicular bone, which are adjacent to the talus, were reconstructed together in a 3D model (Fig. 3) to demarcate the boundary conditions of the distal talus calcaneus and navicular bone that resemble actual ankle-cartilage anatomy. The “sliding contact” condition was added to the interface of the bone considering ankle-cartilage motility, and a “fixed support” condition was added to ensure that the tibia could direct the direction of the loading force. The AnyBody modeling system software (AnyBody Technology, Denmark) was used to set the load conditions. The human body model built in the AnyBody modeling system software was scaled to a height of 168 cm and a weight of 66 kg, and we selected the largest talus compatible with this body model [16, 17]. The reaction force generated in the ankle cartilage was inferred based on the gait cycle, as demonstrated in Fig. 4. The peak point load value and ankle motion angle at the corresponding reaction force were used as variables during topology optimization. The reaction force according to the point in the gait cycle portrayed in the graph may differ from the true reaction force experienced. However, since our purpose was to observe the reaction force trends depending on the gait cycle for selection of the peak point, the difference with the true reaction force experienced by the patient would not affect the results of this study [18–20]. Moreover, the safety of the rational scaffolds and topologically optimized scaffolds formed according to the corresponding reaction force were verified using a finite element analysis (FEA). The model was applied to deduce the reaction force used a 3D-reconstructed talus and adjacent bones (distal tibia, calcaneus, and navicular bone), and the same was also used for topology optimization. In the reaction-force graph, foot-flat, mid-stance, and heel-off positions, which correspond to both maximum and minimum points, were selected as the main phases of the gait cycle [21]. The load size was set as the reaction force in the gait cycle, and the load direction was deduced using tibial alignment at the foot-flat, mid-stance, and heel-off stages and by applying “plantar 10°” (P10), “dorsi 5°” (D5), and “dorsi 10°” (D10), respectively (Fig. 5). The topology optimization analysis module included a “minimize mass” module that has a loading force control function among the safety factor-based “minimize mass” and volume-based “maximize stiffness” conditions. A safety factor of 0.8 was selected. Generally, when considering lightweight, a safety factor of 1.25 is applied. However, because the implant with topology optimization design has parts of the inner structure that are combined with the outer shell structure, which strengthens the mechanical properties, a safety factor of 0.8, which is 64% of 1.25, was applied [22].

### Selecting a simplified rational model for the inner structure

**Fig. 6:**
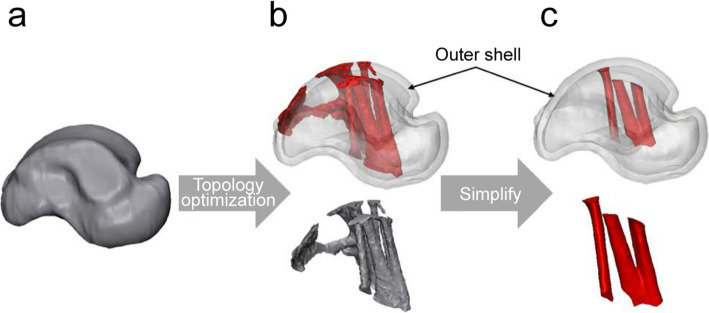
Total talus implant design process. (**a**) Bone model, (**b**) topologically optimized model, and (**c**) simplified rational model

To use the structure deduced by the topologically optimized design as the final product, simplification and standardization processes are required. In this study, the constructs overlapping with the outer shell or the constructs that have no effect on the loading force were eliminated (Fig. 6), and a simplification process was performed to smoothen the surface. Moreover, the the inner structures obtained through simplification were selected as the standard inner structure model for each size.

### Worst-case specification and range selection

To select the worst-case among the P10, D5, and D10 positions for each small, medium, and large size specification (total nine ranges), FEA was performed on each standard model of the representative specification. ANSYS Workbench 2020 R1 software (ANSYS, US) was used to establish a 1.0-mm sized tetrahedron element (Table 3), and the mechanical properties of the cobalt-chromium alloy was calculated (Table 4). For the mechanical properties of the implant material, an alloy comprised of cobalt-28, chromium-6, and molybdenum was applied, and the rigid body setting was applied to the bone model adjacent to the talus [23]. The FEA boundary and loading conditions were set to match topology optimization conditions; however, the loading size was applied based on the test standard for the mechanical properties of prosthetic cartilage (ISO7206-6) [24]. However, because there are no guidelines on the specifications for test standards for the mechanical properties of prosthetic talus cartilage, and because selecting the worst-case through relative comparison is irrelevant to external forces, we used 5340 N, which is used to test the mechanical properties of the femoral stem and neck [24]. To evaluate the effectiveness of the standard model for the inner scaffold in the total talus replacement implants among the talus models of minimum, medium, and maximum sizes in which the inner scaffolds are formed, the worst-case model was selected based on the result of FEA. The peak von Mises stress (PVMS) was compared among the nine models in the three different sizes, and the model with the highest stress was selected as the worst-case.

**Table 3:** Number of elements in each FEA model

FE model (Finite element)	No. of elements
Small (minimum model)	125,278
Medium (medium model)	248,336
Large (maximum model)	271,104

**Table 4:** Mechanical properties of the material

Material	Density (kg/m^3^)	Poisson’s ratio	Elastic modulus (GPa)	Yield strength (MPa)
CoCr alloy	8768	0.29	283	928

#### Evaluation of the effectiveness of the standard model

The talus model selected from the small size range (length, 48 mm; width, 41 mm; and height, 28 mm) with the rational inner-structure-implemented model and that with the topology optimization-design-implemented model were evaluated for relative safety and effectiveness. We used the rational model to adjust the scale of the inner structure of the small size model to correspond to the actual ankle size, which simplified the design process by eliminating the topology optimization design process. For the topologically optimized model, we performed the topology optimization design directly on the ankle to construct the inner structure, which could implement an appropriate inner structure for the individual’s ankle condition and loading distribution.

**Fig. 7:**
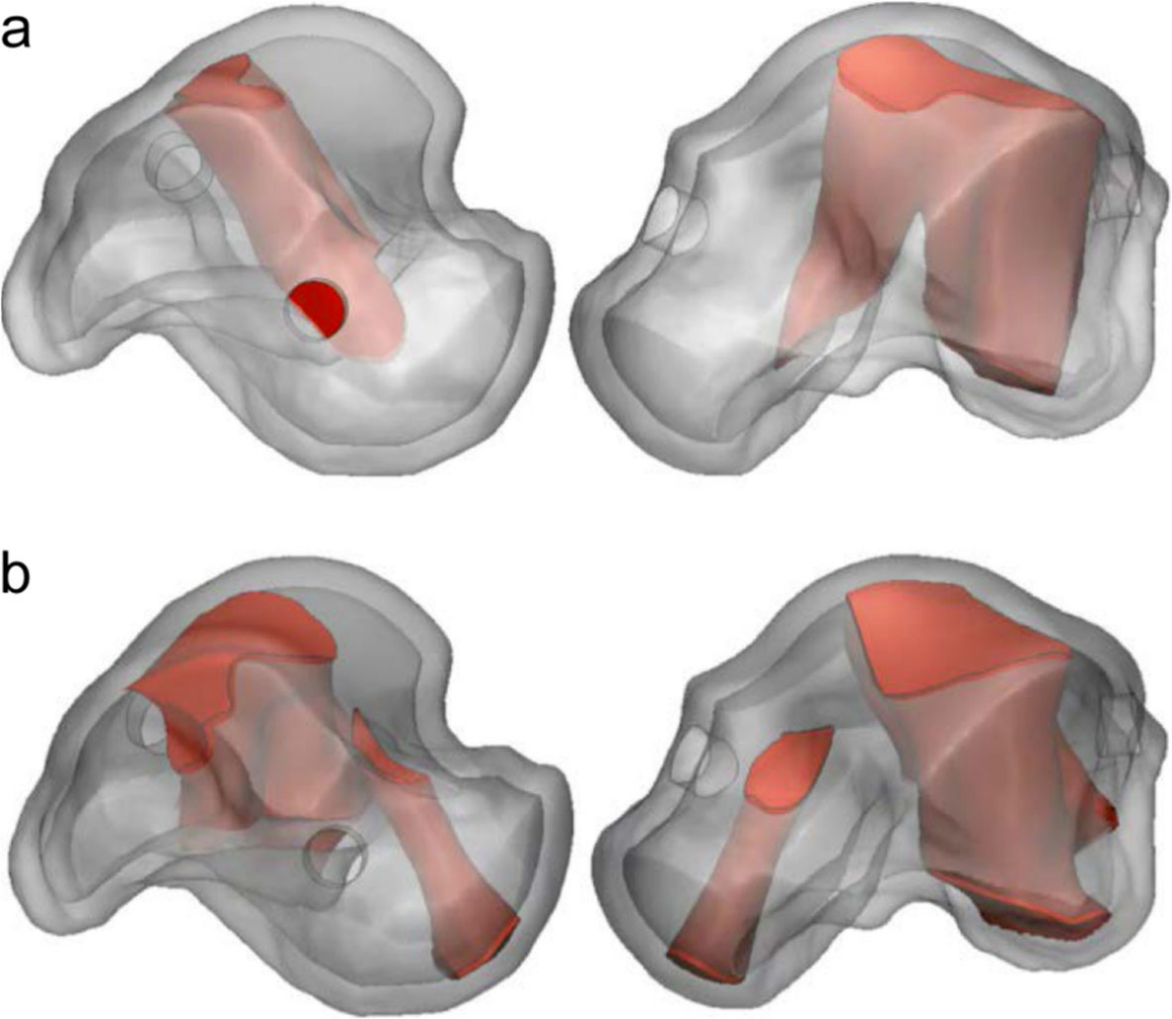
Total talus implant-design-validation model. (**a**) Rational model and (**b**) topologically optimized model

In this study, we performed the validation of a standard model using a bone model selected as the worst-case scenario (length, 48 mm; width, 41 mm; and height, 29 mm), and the rational and topologically optimized models were established (Fig. 7). The constructed models were assessed for the von Mises stress of the FEA using the same boundary conditions. A load of 5340 N was used to select the worst-case model, and these values were compared to evaluate the effectiveness of the standard models.

#### Validation of the total talus implant using a finite-element model

In this study, the convergence of a finite-element model was evaluated based on the error rate of the interpretation of the mesh factor number to evaluate the accuracy of worst-case specifications and standard model efficacy. The convergence item in the Ansys software program was used for evaluation. Accordingly, the “convergence” function was used with an “adaptive mesh refinement” approach, which involves repeated interpretations as the number of mesh factors increases. In the repeated interpretation process, we used the ratio of factors that slowly increased with the “refinement depth 1/2’” setting and an acceptable percentage of chance for convergence set at 5% to evaluate each finite-element model’s errors [25].

## Results

### Standard model for each specification range

**Fig. 8:**
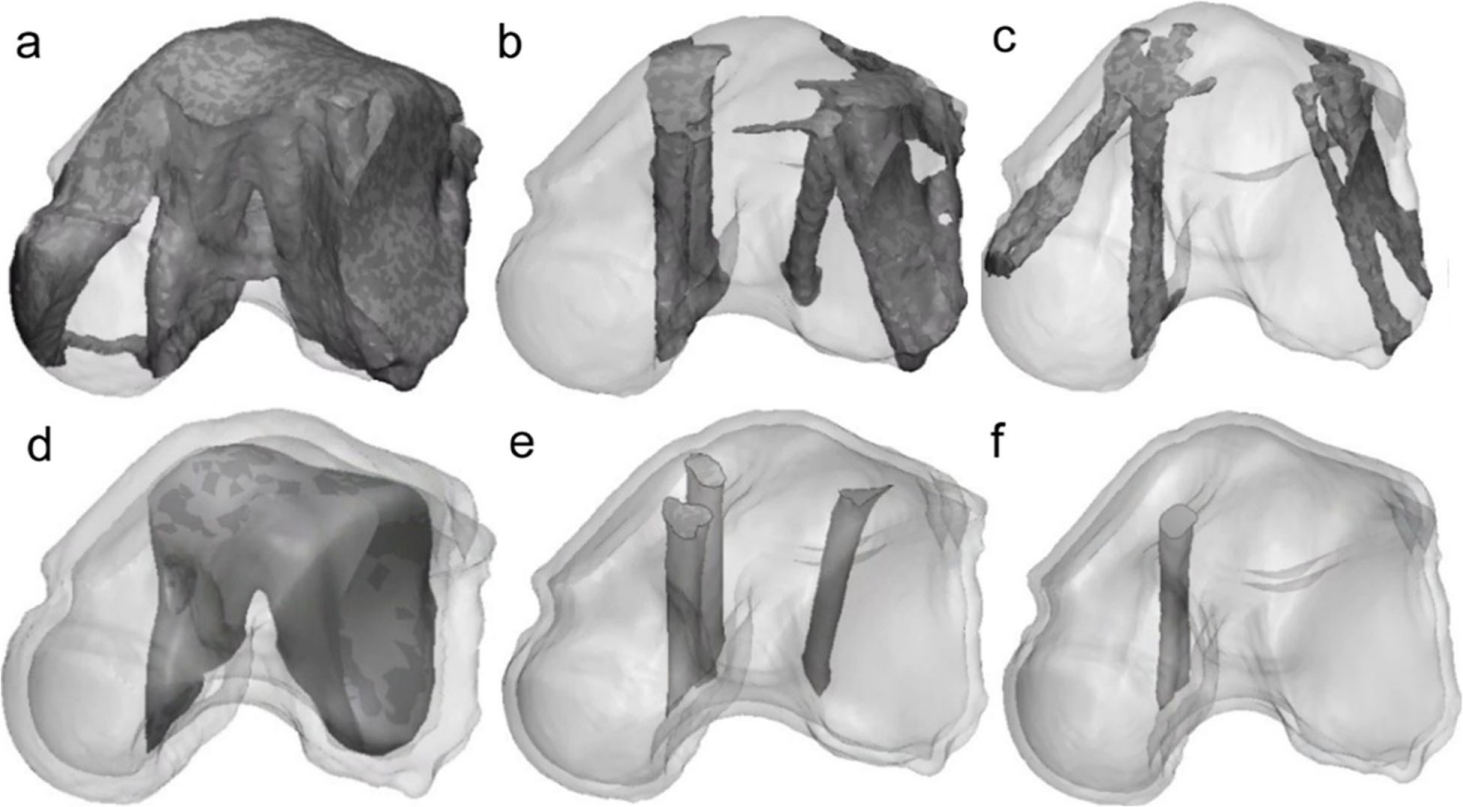
Topology optimization (TO) results and simplified (S) model of each representative specification. (**a**) minimum TO model, (**b**) medium TO model, (**c**) maximum TO model, (**d**) minimum S model, (**e**) medium S model, and (**f**) maximum S model

The standard model for each specification range was deduced based on the minimum, medium, and maximum specifications of the talus model and ankle cartilage anatomical conditions (Fig. 8). The inner scaffold area, to which most loading is transferred, was examined with the topology optimization design. According to this process, the inner scaffold combined model was formed to withstand the peak point load of the gait cycle. Thus, the final model, standardized for each specification’s inner scaffold, was designed with a simplification process. The simplification process involves removing the substantial scaffold, excluding the main passage through which the load must be transmitted, and cleaning the internal structure so that it can be composed of only the necessary structures (Fig. 6). By combining the final design of the inner scaffold of each specification and the initially designed outer shell structure, the representative model for each specification range was designed to select the worst-case model. The weight of each model decreased after applying the rational scaffold, from 1106 g to 965.4 g for the minimum model, from 2534 g to 1524 g for the medium model, and from 8306 g to 3352 g for the maximum model.

### FEA result for worst-case selection

**Fig. 9:**
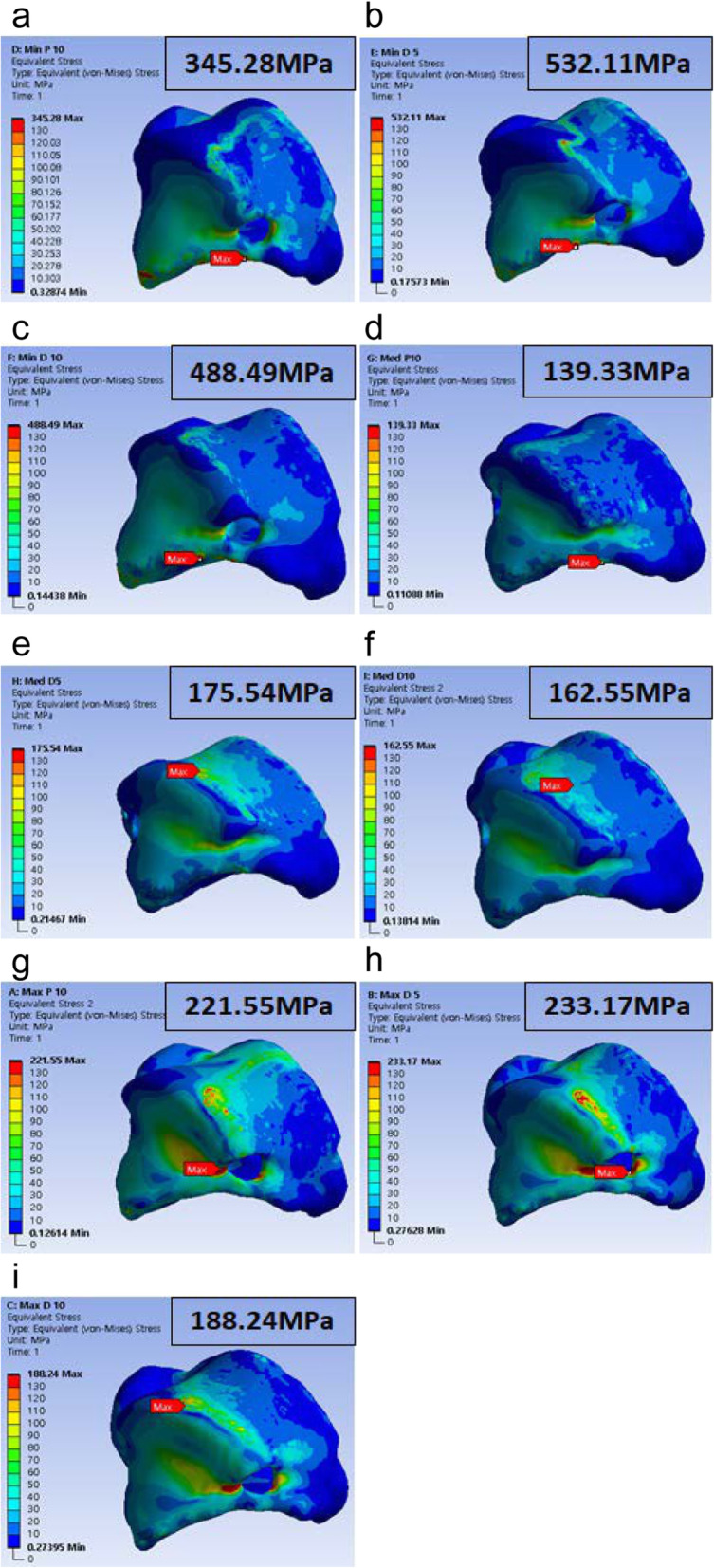
Finite element analysis results for the worst-case specification. (**a**) Minimum plantar 10°, (**b**) minimum dorsi 5°, (**c**) minimum dorsi 10°, (**d**) medium plantar 10°, (**e**) medium dorsi 5°, (**f**) medium dorsi 10°, (**g**) maximum plantar 10°, (**h**) maximum dorsi 5°, and (**i**) maximum dorsi 10°

The FEA result for each model was calculated as shown in Fig. 9. The highest PVMS was 532.11 MPa and was found in the minimum D5 model. In all alignments, the minimum model showed a higher PVMS than in other specification models under the same load. Therefore, the standard model used for the minimum model was selected as the worst-case model and was used to compare the effectiveness analysis of the standard model applied to the rational scaffold to that reflected to the topologically optimized model for each size.

### Results of the analysis for validity evaluation of the standard model

**Fig. 10:**
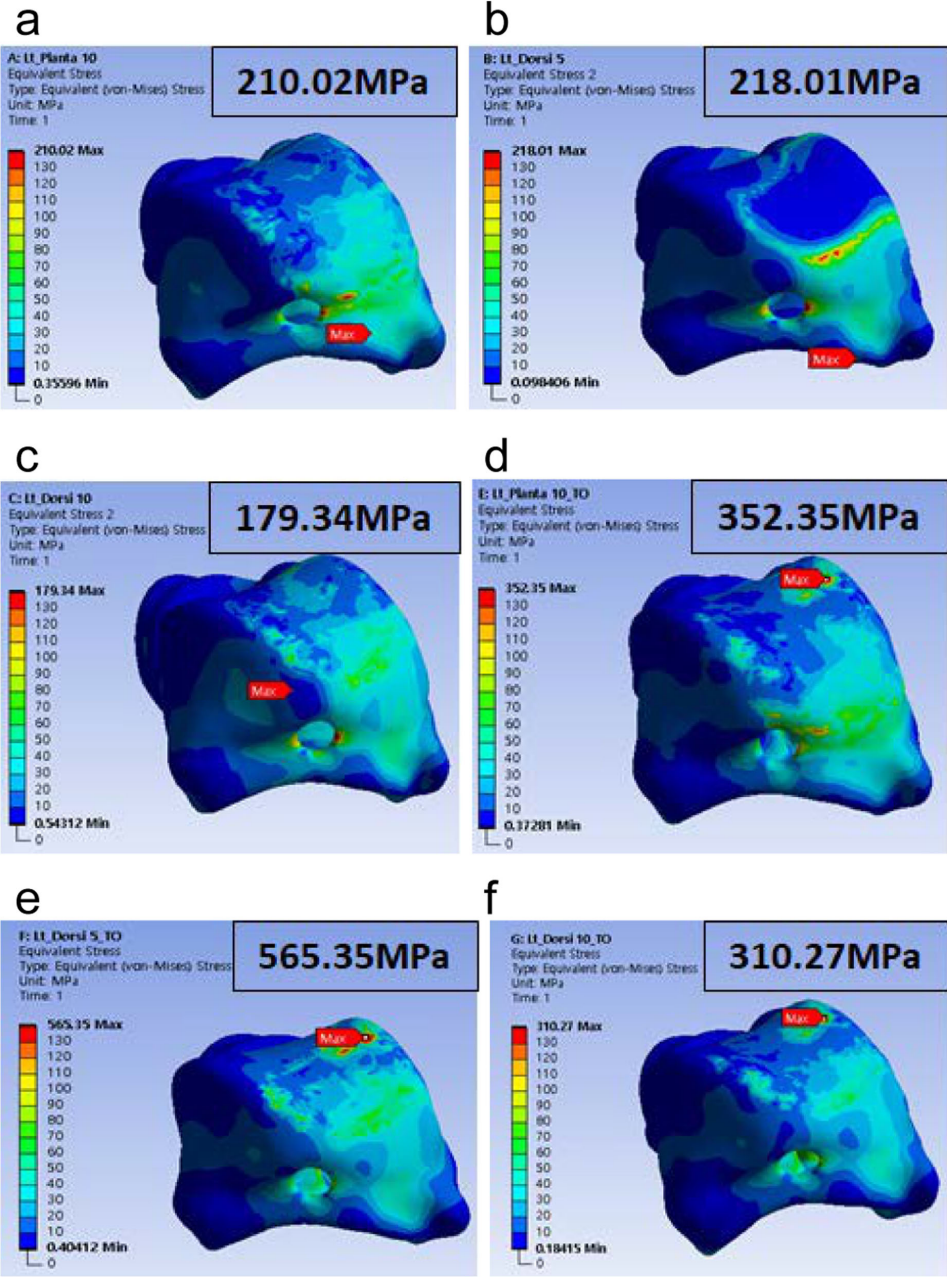
Finite element analysis results for the validation of the rational model. (**a**) Rational model plantar 10°, (**b**) rational model dorsi 5°, (**c**) rational model dorsi 10°, (**d**) TO model plantar 10°, (**e**) TO model dorsi 5°, and (**f**) TO model dorsi 10°

Results of the FEA of each model are displayed in Fig. 10. The analysis results demonstrated that in the three types of tibial alignment (P10, D5, and D10) rational models, PVMSs were 210.02 MPa, 218.01 MPa, and 179.34 MPa, respectively. The PVMSs for the topologically optimized models were 352.35 MPa, 565.35 MPa, and 310.27 MPa for P10, D5, and D10, respectively. A higher PVMS was observed in the topologically optimized model, regardless of the tibial alignment throughout the gait cycle.

This confirmed that the implant using the model based on the standard inner structure had a lower PVMS under the same load compared to the implant based on the topologically optimized model, which was structurally safer. Additionally, the analysis result of the minimum talus implant using a model based on the standard inner structure showed a lower value than the yield strength (928 MPa) of the CoCr alloy (ASTM F75) material; accordingly, the inner scaffold implemented according to the size range was applied to the actual talus model, and it was confirmed that the rational inner scaffold model was effective to withstand the load enacted by the human body.

#### Finite-element model validation

To validate the finite-element model used for assessing the effectiveness of the standard model for each specification and for selecting the worst-case, we performed convergence evaluation, in which we assessed the change in PVMS with an increase in the number of elements. The error rate of PVMS change for each model in adaptive mesh refinement convergence was confirmed to be 0.34–4.68%. Therefore, this confirms that the maximum error rate for each model element is within 5%.

## Discussion

To assess the effectiveness of the standard implant model for total talar replacement, a “rational model” and a “topologically optimized model” were designed for an identical talus, and FEA was performed to evaluate their mechanical properties.

To validate the finite-element model used in the research, convergence was assessed using an adaptive mesh refinement method in which the error rate of the repeated analysis with an increasing number of elements was calculated. This error rate was 0.34–4.68%, which was below the initially set maximum tolerated error rate of 5%, and this confirmed the validity of the model used in this research.

In this study, the worst-case model for the representative standard model of each specification range was determined as the minimum model. In the FEA result of the rational and topologically optimized models, based on different design methods, the rational model showed a PVMS of 218.01 MPa at D5, which was lower than the PVMS of the topologically optimized model at D5 (565.35 MPa). This result confirmed that the rational inner-scaffold-design method proposed in this study is relatively safe and that the three types of rational-model scaffold (minimum, medium, and maximum) can cover all anatomical sizes of talus discussed in this study, which demonstrated their validity for total talus replacement design. Moreover, as the rational scaffold was applied, the weight was reduced, but safety was ensured to withstand approximately five times the weight of an adult male. Therefore, we believe that, in future design processes of total talus replacement implants, the rational-model inner scaffold can be used instead of applying a topology optimization design for each product, to reduce design time and manufacture mechanically safer implants. Moreover, we believe that, with the design elements proposed in this research, designing implants that meet the clinical design requirements of mechanical stability is possible.

The load strength used for FEA was 5340 N, which is the load strength used to test the mechanical properties of the femoral stem, a prosthetic hip component. This was because test standards for a prosthetic talus cartilage test are inadequate. Although this can be viewed as a limitation of the study, it did not have a marked impact on the aim of this study, which focused on comparing the safety and assessing the effectiveness of the rational and topologically optimized models. Instead of assessing the mechanical safety of the standard model for each specification, a relative comparison was performed with the worst-case model to evaluate effectiveness. Therefore, it is difficult to confirm the mechanical safety of the total talus replacement implant based on FEA in this study, and for this purpose, a standard for mechanical property assessment needs to be established with assessments performed on a manufactured implant.

## Conclusion

Subsequent research is needed to determine which implant is actually safer, and it is necessary to manufacture an actual product to test its mechanical performance. The results will vary depending on many factors, such as the patient’s weight, bone condition, osteoporosis status, lifestyle, and age. However, according to the results of the analytical approach used in this study, the standard model to which the rational scaffold was applied was deemed safer than the model to which the topologically optimized scaffold was applied. The three types of rational scaffold, could cover most of the talus within the anatomical dimension, which is necessary for total talar replacement. In the future, an effective total talar replacement design with a reduced design time may be possible by applying a rational inner scaffold without performing topologically optimized design for each patient’s talus replacement. We believe that our implant design process will meet clinical design requirements, such as ensuring customization, yielding a lightweight implant, and ensuring mechanical safety.

## Data Availability

Not applicable.

## Abbreviations

3D: Three-dimensional
AVN: Avascular necrosis
FEA: Finite element analysis
PVMS: Peak von-Mises stress
TO: Topology optimization
P10: Plantar 10°
D5: Dorsi 5°
D10: Dorsi 10°
